# Risk factors for urinary tract infection in children with urinary urgency

**DOI:** 10.1590/S1677-5538.IBJU.2017.0434

**Published:** 2018

**Authors:** Rhaiana Gondim, Roberta Azevedo, Ana Aparecida Nascimento Martinelli Braga, Maria Luiza Veiga, Ubirajara Barroso

**Affiliations:** 1 Centro Pediátrico de Distúrbios Urinários (CEDIMI), Salvador, BA, Brasil; 2 Departamento de Urologia, Unidade de Urologia Pediátrica, Faculdade de Medicina e Saúde Pública da Bahia, Universidade Federal da Bahia, Salvador, BA, Brasil

**Keywords:** Urinary Tract Infections, Lower Urinary Tract Symptoms, Urinary Bladder, Overactive

## Abstract

**Purpose:**

To identify which independent variable would be strong predictor of febrile urinary tract infection (UTI) in children and adolescents with overactive bladder.

**Materials and Methods:**

A search was made of the institute's database for all patients diagnosed with overactive bladder over the preceding four years. Children and adolescents under 18 years of age with overactive bladder and no neurological or anatomical alterations of the lower urinary tract were included in the study. The independent variables were: sex, age, ethnicity (Brazilians of African descendence/others), the presence of urinary urgency, daytime incontinence, enuresis, frequent urination, infrequent voiding (≤3 voids/day), nocturia, holding maneuvers, straining to void, intermittent urinary flow, constipation and encopresis. An analysis was conducted to identify patients with febrile UTI and subsequently determine predictors of this condition. Univariate and multivariate analyses were performed.

**Results:**

Overall, 326 patients (214 girls/112 boys) were evaluated. The mean age of the patients was 7.7±3.19 years (± standard deviation). The incidence of febrile UTI was 39.2%. Being female and infrequent voiding were factors significantly associated with febrile UTI, both in the univariate and multivariate analyses.

**Conclusions:**

These results show that being female and infrequent voiding constituted significant risk factors for a diagnosis of febrile UTI in these children.

## INTRODUCTION

Lower urinary tract symptoms (LUTS) in children are characterized by urgency to void, daytime incontinence, holding maneuvers and an increase or decrease in the frequency of urination in the absence of any neurological disease or anatomical abnormality of the lower urinary tract (1). Daytime incontinence is a common finding in children, with a reported prevalence rate of 6% in 7-year old girls (2). As well as the frequency of these symptoms, LUTS also represent a common cause of urinary tract infection (UTI) in toilet-trained children, which, in addition to the accompanying bothersome symptoms, may also result in renal scarring, hypertension and diminished renal function (3-5).

Some risk factors for UTI have already been identified, including sex, ethnicity, vesicoureteral reflux, neurogenic bladder, phimosis, anatomical abnormalities of the lower urinary tract, constipation and the presence of LUTS (6, 7). Some LUTS such as urinary retention, elevated residual urine volume, infrequent voiding and voiding postponement have been reported to be associated with UTI (8-11). Overactive bladder is the most common lower urinary tract disorder; however, its association with UTI remains to be clarified. The objectives of the present study were to evaluate the incidence of UTIs in children with isolated overactive bladder and identify the possible predictors of UTI.

## MATERIALS AND METHODS

This is a cross-sectional study of children and adolescents diagnosed with urinary urgency as defined by the International Children's Continence Society (ICCS). A search was made in the institute's database for all patients diagnosed with LUTS over the preceding four years. A structured questionnaire on urinary symptoms, the Dysfunctional Voiding Scoring System (DVSS) and the Rome III questionnaire for constipation were completed for each child. A history of UTI was investigated by asking the children's caregivers about any sudden appearance of fever or symptoms such as incontinence, dysuria, urinary urgency and frequent urination. Any findings of >10^5^ colony-forming units (CFU)/mL in cultures from urinary samples collected by catheter in small children and from midstream urine specimens in older ones were taken into consideration. Only the cases of UTI confirmed by culture were considered for inclusion in the sub-analysis of infection. All children diagnosed with a UTI were treated with a course of antibiotics of 5-7 days in the absence of fever or 10-14 days if fever was present. Urgency was considered indicative of an overactive bladder only if no UTI was present.

Children and adolescents under 18 years of age with urinary urgency and no neurological disease or anatomical abnormality of the lower urinary tract, with post-void residual urine volume <10% of the expected bladder capacity as shown by ultrasound, and a bell-shaped or tower-shaped urinary flow curve were admitted to the study. Consequently, patients with dysfunctional voiding, in whom the urinary flow curve pattern is staccato, were excluded from the study, with only those with an isolated overactive bladder being included. In addition, children whose medical records lacked sufficient data were also excluded.

The independent variables consisted of sex (male/female), the presence of daytime incontinence, nocturnal enuresis (at least twice a week), frequent urination, infrequent voiding (≤3 voids/day), nocturia, holding maneuvers, straining to void, intermittent urinary flow, vaginal discharge, constipation and encopresis. Constipation was diagnosed by the presence of less than three bowel movements per week with abdominal pain and straining at defecation. Encopresis was defined as the involuntary leakage of stool in children over four years of age (12). Vaginal discharge was determined as being present if reported in the questionnaire, with no laboratory examination being performed for confirmation of diagnosis. The dependent variable was the presence of febrile UTI.

A univariate analysis was performed to evaluate factors predictive of febrile urinary tract infection. The chi-square test was used in the bivariate analysis, with any variable that achieved statistical significance (p<0.02) then being included in the multivariate analysis. Statistical significance at the multivariate analysis was defined as p<0.05.

The internal review board of the *Escola Bahiana de Medicina* approved the study protocol.

## RESULTS

A total of 326 children (214 girls/112 boys) with a mean age of 7.7±3.19 years (± standard deviation [SD]) (range 2-16 years) were admitted to the study. Six children under three years of age were included because they had a UTI and clear symptoms of urinary urgency. In this study, the presence of symptoms that have often been associated with UTIs was confirmed, including holding maneuvers (73.9%), seen in patients with postponement behavior, enuresis (68.9%), urge incontinence (85%) and constipation (76.5%). Although vaginal discharge and infrequent voiding have been frequently reported as being associated with UTIs, the rate of vaginal discharge in the present sample was no higher than 28% (56/202 girls) and the rate of infrequent voiding was as low as 12% (38/323 boys and girls). Table-1 shows the distribution of the LUTS found in the sample. The incidence of febrile UTI was 39.2%.

**Table 1:** Distribution of lower urinary tract symptoms in the sample population.

Symptoms	Present		Absent	
	n	%	n	%
Holding maneuvers	229	73.9	81	26.1
Enuresis	226	68.9	101	30.8
Suprapubic pain	183	58.5	130	41.5
Urge incontinence	278	85	49	15
Urinary frequency	176	55	144	45
Urinary incontinence	148	46	174	54
Constipation	202	76.5	62	23.5
Nocturia	94	29	230	71
Stress incontinence	88	27.3	234	72.7
Giggle incontinence	102	32.1	216	67.9
Vaginal discharge	56	27.7	146	72.3
Straining to void	101	31.1	224	68.9
Curtsy sign	81	37.5	135	62.5
Infrequent voiding	38	11.8	285	88.2

As shown in Table-2, infrequent voiding (p=0.05) and being female (p=0.03) were found to represent risk factors for febrile UTIs. Many of the children experienced symptoms that are very common in lower urinary tract dysfunction such as dysfunctional voiding, frequent urination, suprapubic pain and constipation; however, no statistically significant association was found between those symptoms and febrile UTI.

**Table 2:** Factors associated with febrile urinary tract infection in patients with overactive bladder: univariate analysis.

Factors		Urinary tract infection		No urinary tract infection		p-value	OR	95%CI
		n	%	n	%			
Sex	Male	32	34.8	60	65.2	0.031	1.801	1.080-3.003
	Female	97	49	101	51			
Enuresis	Yes	88	53	109	55.3	0.900	1.063	0.649-1.742
	No	41	43.2	54	56.8			
Holding maneuvers	Yes	96	46.6	110	53.4	0.097	1.641	0.940-2.864
	No	25	34.7	47	65.3			
Nocturia	Yes	32	40	48	60	0.429	0.800	0.474-1.351
	No	114	54.5	95	45.5			
Straining to void	Yes	46	49.5	47	50.5	0.208	1.385	0.844-2.272
	No	82	41.4	116	58.6			
Urinary incontinence	Yes	63	47	71	53	0.476	1.215	0.762-1.937
	No	65	42.2	89	57.8			
Infrequent voiding	Yes	22	59.5	15	40.5	0.050	2.073	1.027-4.186
	No	104	41.4	147	58.6			
Frequent urination	Yes	63	41.7	88	58.3	0.283	0.760	0.476-1.214
	No	65	48.5	69	51.5			
Suprapubic pain	Yes	78	47	88	53	0.223	1.359	0.838-2.204
	No	45	39.5	69	60.5			
Vaginal discharge	Yes	28	51.9	26	48.1	0.748	1.143	0.607-2.151
	No	65	48.5	69	51.5			
Curtsy sign	Yes	43	55.8	34	44	0.113	1.610	0.909-2.852
	No	55	44	70	56			
Encopresis	Yes	4	28.6	101	71.4	0.259	0.432	0.129-1.447
	No	63	48.1	68	51.9			
Constipation	Yes	85	46.2	99	53.8	0.742	1.159	0.607-1.751
	No	20	42.6	27	58.4			
Ethnicity	African descent	70	45.2	46	54.1	1.0	1.030	0.605-1.751
	Other	39	45.9	85	54.8			

**OR =** odds ratio; **95%CI =** 95% confidence interval.

Being female and infrequent voiding were factors that remained significantly associated with febrile UTI in the multivariate analysis and are therefore considered to constitute independent risk factors for this condition (Table-3).

**Table 3:** Febrile urinary tract infection in children and adolescents with overactive bladder: multivariate analysis.

Predictive factor	p-value*	95%CI
Being female	0.024	0.019-0.265
Infrequent voiding	0.05	0.008-0.334

* p-value for the multivariate analysis;

**95%CI =** 95% confidence interval

## DISCUSSION

In this study, being female and infrequent voiding were found to constitute independent risk factors for febrile UTI. The association between UTI and sex has been reported previously (13). The rate of UTI in the first three months of life has been reported as 7.5% in girls, 2.4% in circumcised boys and 20.1% in uncircumcised boys. In the first year of life, UTIs are more common in boys (3.7%) compared to girls (2%). In pre-pubertal girls and boys, the incidence of UTIs is 3% and 1%, respectively (13). Recent reviews have reported findings of *E. coli* in 96% of girls and in 89% of boys presenting with a UTI. Other infections commonly present include, in decreasing order of frequency, *Klebsiella, Proteus, Enterococcus* and *Pseudomonas* (13, 14).

Infrequent voiding was shown to be a risk factor for febrile UTI both in the univariate and multivariate analyses, and can be regarded as an independent predictor. This symptom could lead to the development of a UTI by facilitating the accumulation of residual urine, a known risk factor for urinary infection. Girls are more likely to postpone voiding compared to boys and this may explain the finding of sex as an independent predictor of febrile UTI; nevertheless, voiding postponement as a risk factor failed to reach statistical significance.

Some factors did not correlate positively with a febrile UTI although they had been expected to do so. These included vaginal discharge, holding maneuvers and constipation. Vaginal discharge, the so-called *nonspecific vulvovaginitis*, has been linked to inadequate personal hygiene, particularly following defecation. Vulvovaginitis is often associated with LUTS. Despite this apparent association, no studies reporting this finding in children with LUTS were identified. The present study was unable to confirm whether the vaginal discharge was of bacterial or fungal origin, since no laboratory tests or culture were performed; therefore, these data must be interpreted with extreme caution.

The incidence of UTI in children with overactive bladder may be higher in those who usually perform holding maneuvers to postpone voiding compared to those who do not use these maneuvers (2, 15). In the present study, the association between the practice of holding maneuvers and UTI did not achieve statistical significance (p=0.097; OR = 1.64; 95%CI: 0.940 - 2.864). Holding maneuvers could redirect urine from the urethra into the bladder, increasing urinary stasis and predisposing the child to recurrent UTI. Straining to void can lead to hypertrophy of the bladder wall and decreased perfusion of the detrusor (15), which could lead to a reduction of the specific factors in the bladder that protect against UTI. Nevertheless, no statistically significant association was found between this symptom and UTI in the present study.

A strong association has been reported in the literature between constipation and LUTS. In fact, constipation must be considered as three different entities: constipation alone, encopresis alone and both conditions together. Some investigators have already linked dysfunctional voiding with constipation (p<0.05) (12). This link has been widely studied, with previous studies showing a higher incidence of bowel dysfunction, including constipation (39%), in patients with dysfunctional voiding (12). Interestingly, constipation was not found to be a predictor of UTI in the present sample. In children with encopresis, severe urinary urgency has often been reported, with the condition often disappearing following initiation of anticholinergic therapy even before the urgency has fully subsided (16, 17). The relationship between fecal and urinary retention is well known; therefore, in patients with LUTS, constipation could play a role in the rate of UTI.

There are some limitations associated with this study, since it may be underpowered for some parts of the analysis. The urine culture results confirming the presence of a UTI were not available in all cases, with undocumented information provided by the child's parents being used in such cases. Therefore, some errors in the diagnosis of UTI may have occurred in the study. Since the Rome III criteria were used to diagnose constipation, the data on this symptom must be evaluated with caution.

## CONCLUSIONS

Being female and infrequent voiding were found to constitute important risk factors for febrile UTI in children with overactive bladder. In children with these risk factors, LUTS should be treated aggressively. In addition, these children need to be monitored more closely and may benefit from prophylactic antibiotic treatment.
